# A Non-Destructive Testing Method for Fault Detection of Substation Grounding Grids

**DOI:** 10.3390/s19092046

**Published:** 2019-05-02

**Authors:** Xiujuan Wang, Zhihong Fu, Yao Wang, Renkuan Liu, Lin Chen

**Affiliations:** 1 School of Electrical Engineering, Chongqing University, State Key Laboratory of Power Transmission Equipment and System Security, Chongqing 400044, China; shanshui510@163.com (X.W.); 20161101003@cqu.edu.cn (Y.W.); 20161113067@cqu.edu.cn (R.L.); 20161101018@cqu.edu.cn (L.C.); 2 Chongqing Triloop Prospecting Technology Co. Ltd., Chongqing 402660, China

**Keywords:** grounding grid, conductor breakpoint detection, magnetic source excitation, non-destructive electromagnetic detection

## Abstract

The grounding grid is critical to the safety and stability of a power system. Corrosive cracking of the grounding conductor is the main cause of deterioration of grounding grid performance. Existing fault diagnosis methods for grounding grids are limited by the number and distribution of grounding leads, and some of them cannot be used for online detection. This paper proposes a grounding grid detection method based on magnetic source excitation. The measuring device consists of four coils, two horizontal excitation coils, and two vertical receiving coils. The secondary magnetic field signal is extracted from the primary field and the background field by properly positioning the coils, such that the measured signal can reflect the underground media more accurately. The measuring device of the method is portable, the measurement process is contactless with the grounding grid, and it is not limited by the grounding leads. Furthermore, it has a strong anti-interference ability and can realize online detection. It was proven by simulations and experiments that the proposed method has a higher measurement accuracy and stronger anti-interference ability when compared with existing methods. This paper also discusses the influence of various factors such as the number and the location of the breakpoints, the frequency of the excitation source, the soil resistivity, and stratification from the measurement data. It was proven that the method has high precision and a wide application range, and is important for guiding significance and reference value in engineering applications.

## 1. Introduction

Grounding grids are designed and installed to make power systems safe. The performance of grounding grids has to meet the requirements from utilities during the entire operation time. Many factors such as corrosion, the electromotive forces of the grounding current, and loss of grounding rods may damage the grounding grids, and eventually lead to performance degradation [1,2]. In such cases, the performance of the grounding grid will not meet the specified standards, which will directly endanger the normal operation of the power system and the safety of the workers. Therefore, the power industry attaches great importance to the performance of the grounding grid [3]. In order to ensure the normal operation of the power system and avoid blind excavation, the detection of the performance status of the grounding grid has become an important research topic.

According to a survey conducted by the IEEE/PES Substations West Coast Subcommittee [4], almost all substations surveyed had evaluated the integrity of their grounding grids at some point. The high-current method recommended by the IEEE can only be carried out when the power system is under outage and the outcome only reflects the connectivity of the grounding leads and the main grid [1]. Currently, monitoring and evaluating the grounding grid conditions rely on electrical parameters obtained from measurements, including the ground resistance, the touch voltage, and the step voltage [5,6,7,8,9,10,11]. Most of the parameter measurement methods are offline. An online touch-and-step voltage measuring scheme, which continuously monitors the condition of the grounding grids, was proposed by Xu [3]. However, sufficient grounding leads are needed to monitor and evaluate the grounding grid condition. This method cannot be used in measured areas where the required potential and current electrodes are not present. Furthermore, as soil resistivity is affected by seasonal changes and other factors [12,13], the measured results of regular parameters do not reflect the actual situation of the grounding grid. In the existing grounding grid detection method, a direct current in injected into the grounding grid as an excitation and measurements are interpreted based on electric network theory [14,15]. For the electromagnetic field-based method, a harmonic or square wave current is injected into the grounding grid as an excitation [16,17,18]. These methods require as many grounding leads as possible to improve the measurement accuracy. We propose a four-coil structure detection method that can overcome these drawbacks.

In Reference [19], a three coil metal detector consisting of an excitation coil and two receiving coils was proposed for cable detection. The coil structure was optimized and a detailed theoretical derivation and experimental analysis were given. The proposed method had an important guiding significance for this paper. This paper proposes a grounding grid detection method based on magnetic source excitation. The measuring device consists of four coils, the measurement process is contactless with the grounding grid, and it is not limited by the grounding leads. Simulations of the proposed method show that the proposed method has a higher measurement accuracy and stronger anti-interference ability compared with the existing methods. Furthermore, this paper analyzed the influence of the number and location of the breakpoints, the frequency of the excitation sources, soil resistivity, and stratification on the measured data, which proved the feasibility of the method in engineering applications.

In Section 2, this paper introduces the theoretical basis and implementation of the proposed four-coil device. In Section 3, the scale and distribution distance of the coils are given by CDEGS grounding grid simulation software. The simulation results demonstrate that the proposed method can locate the grounding conductor and the breakpoint. Compared with the existing method, the four-coil structure has obvious advantages. In Section 4, the possible factors that may influence the grounding grid detection are analyzed, and the feasibility of the four-coil structure is confirmed by CDEGS software simulation. In Section 5, the field testing for a substation shows that the experimental results are consistent with the actual situation. Finally, in Section 6, the proposed method is summarized.

## 2. The Proposed Four-Coil Detection Method

In this paper, a magnetic source detection method for the grounding grid state is proposed. The measurement device is composed of four coils in a specific arrangement. The two horizontally placed coils are excitation coils, and the two vertically placed coils are receiving coils. The measurement result is mainly the secondary field signal, which effectively avoids magnetic field interference and can accurately reflect the actual situation of the grounding conductor [20]. 

### 2.1. Generation of Induced Current in the Grounding Grid Conductor

**Figure 1:**
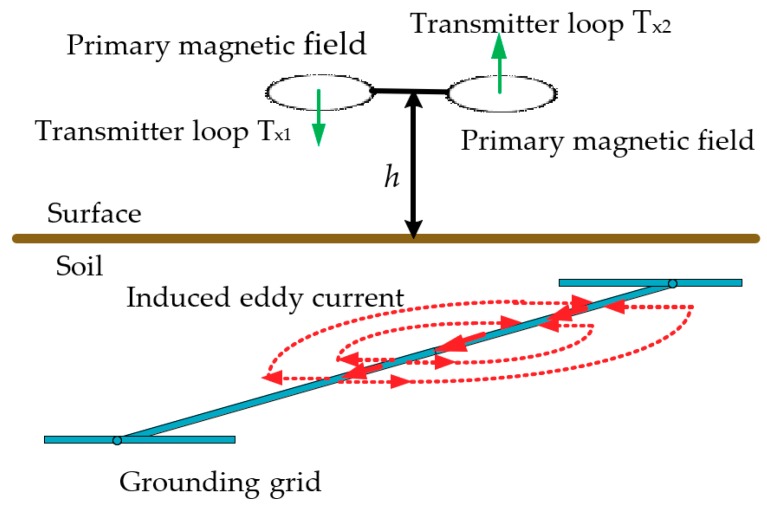
The generation process of magnetic induction.

The generation process of the induced current flowing in the grounding grid conductor is depicted in Figure 1. It can be seen from Figure 1 that the transmitter loops, T_x1_ and T_x2_, are oriented horizontally and placed at the same height *h* above the ground surface. T_x1_ and T_x2_ are driven by an alternating current with the same magnitude but in opposite directions. According to the law of electromagnetic induction and the superposition principle, on the mid vertical plane of the connection between the center points of the two loops, the total magnetic field generated by the two coils is much stronger than that by a single coil.

The induced current is generated in the surrounding space under the excitation of the primary magnetic field. Soil is a nonmagnetic substance and the grounding conductor is a ferromagnetic substance. Additionally, the resistivity of the soil is much higher than that of the grounding conductor. The major portion of the induced current flows along the grounding conductor. Only a very small portion flows into the ground. When the grounding conductors near T_x1_ and T_x2_ are in good condition, the major portion of the induced current flows along the grounding conductor, and only a very small portion flows into the ground. If a conductor segment is broken, no or very little induced current flows in the conductor.

### 2.2. Generation and Detection of the Secondary Magnetic Field

**Figure 2:**
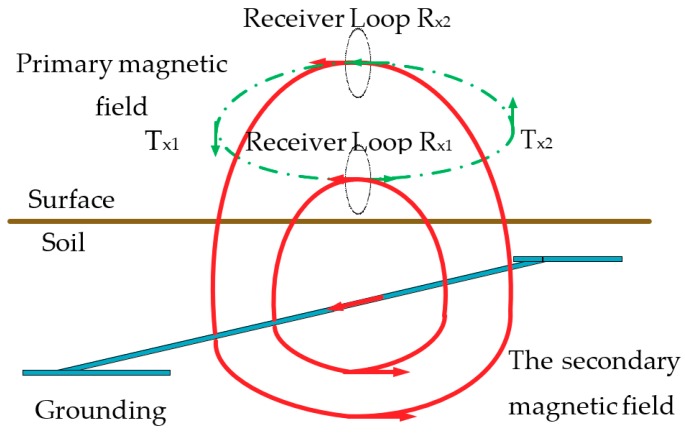
Detection process of the secondary magnetic field.

The generation and detection process of the secondary magnetic field is shown in Figure 2. The induced current flowing in the grounding conductor generates a secondary magnetic field in the surrounding space. The distribution of the secondary magnetic field depends on the condition of the ground conductor. When there is no conductor in the proximity or the conductor is broken, the induced current, and thus the secondary field, are very weak and can be neglected. Thus, the status of the grounding conductor segment can be evaluated by detecting the secondary magnetic field. The magnetic field measured above the ground is the superposition of the primary and secondary magnetic fields. The primary field is much stronger than the secondary, so the information of the secondary magnetic field is masked.

The receiver loops R_x1_ and R_x2_ are oriented vertically and placed right above the desired grounding grid conductor segment, in order to measure the horizontal magnetic field. R_x1_ is lower than the transmitter loops T_x1_ and T_x2_, while R_x2_ is higher. In the proposed method, the interference of the primary magnetic field is eliminated, and the net secondary magnetic field is obtained by using two magnetic receiver loops R_x1_ and R_x2_, which are deliberately arranged.

The magnetic flux densities measured by R_x1_ and R_x2_ can be decomposed into two parts as

(1) $$B_{rx1}=B_{p1}+B_{s1}$$

(2) $$B_{rx2}=B_{p2}+B_{s2},$$

where, $B_{rx1}$ and $B_{rx2}$ are the magnetic flux densities measured by the receiver loops R_x1_ and R_x2_, respectively; likewise, $B_{p1}$, $B_{p2}$, and $B_{s1}$, $B_{s2}$ are the primary field and secondary field components, respectively.

The total magnetic intensity $B_{out}$ can be expressed as

(3) $$B_{out}=B_{rx1}+B_{rx2}=B_{p1}+B_{s1}+B_{p2}+B_{s2}$$

The directions of $B_{p1}$ and $B_{p2}$ are opposite, and their magnitude can be adjusted to the same value by changing the heights of R_x1_ and R_x2_. In this case, $B_{out}$ only includes the net secondary component:

(4) $$B_{out}=B_{s1}+B_{s2}.$$

In this section, the feasibility of the four-coil grounding grid detection structure was theoretically analyzed. The scale and distance of the coils are given in Section 3 by CDEGS grounding grid simulation software.

## 3. CDEGS Software Simulation Verification

The CDEGS simulation software developed by Canadian SES company is the authoritative grounding simulation analysis software as recognized by the power industry [21]. The sizes and spacings of the coils were determined by CDEGS simulations. A typical grounding grid model was built by the simulation software. The applicability of the proposed method was verified by measuring the set area. The simulation results of the proposed measurement method were compared with the current injection method [22,23], which further proves the advantages of the proposed method.

**Figure 3:**
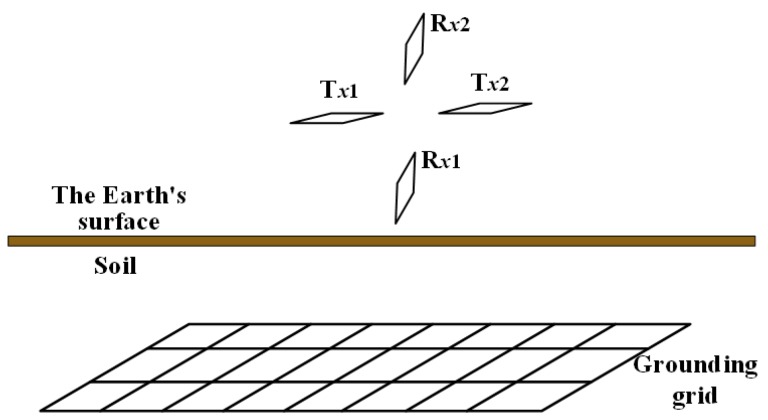
A simulation model of the four-coil structure.

Since the CDEGS simulation software cannot set a circular coil, a square coil with an equivalent side length was used as an approximate replacement, as shown in Figure 3. The transmitter coils (T_x1_, T_x2_) and receiver coils (R_x1_, R_x2_) were square loops, each with a side length of 20 cm and each having 20 turns. Both transmitter coils were placed 0.4 m above the ground surface. The center points of these two coils were 1.4 m apart. The transmitter system was placed in a location without the grounding grid. The position of the two receiving coils was determined by measuring the magnetic flux density on the line perpendicular to the ground and passing through the midpoint of the centerline of the two transmitting coils.

**Figure 4:**
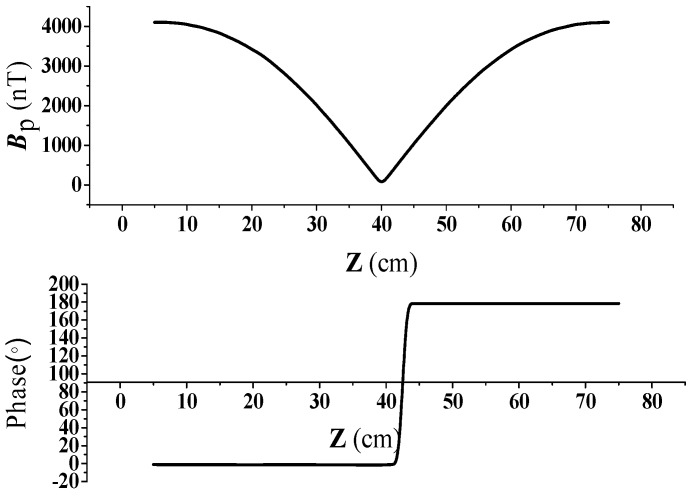
The primary magnetic field profile.

A current with an amplitude of 10 A and a frequency of 50 kHz was injected into the transmitter coils. The measuring line is perpendicular to the ground and passes through the line connecting the midpoints of the two transmitting coils. The *Z*-axis direction was defined to be perpendicular to the ground in a vertical direction, where **Z** = 0 m is the ground surface. The simulation results of the primary magnetic flux $B_{p}$ are shown in Figure 4, which presents the magnitude and phase of the magnetic flux density measured at various distances from the ground surface. It can be seen from Figure 4 that the magnitude of the magnetic flux density is symmetric about the height of the transmitter coils, 0.4 m above the ground. Additionally, the phase changes 180° at that position. It is observed from Figure 4 that when the R_x_ coils are placed at a distance of 0.1 m and 0.7 m above the ground surface, the magnitudes of the primary field from the excitation current are the same, whereas the phases differ by 180°. 

Thus, in the following, we place the receiver coils R_x1_ at a height of 0.1 m and R_x2_ at 0.7 m above the ground surface. The R_x_ coils are coplanar and perpendicular to the plane of the two transmitter coils.

### 3.1. Grounding Grid Simulation Model 

**Figure 5:**
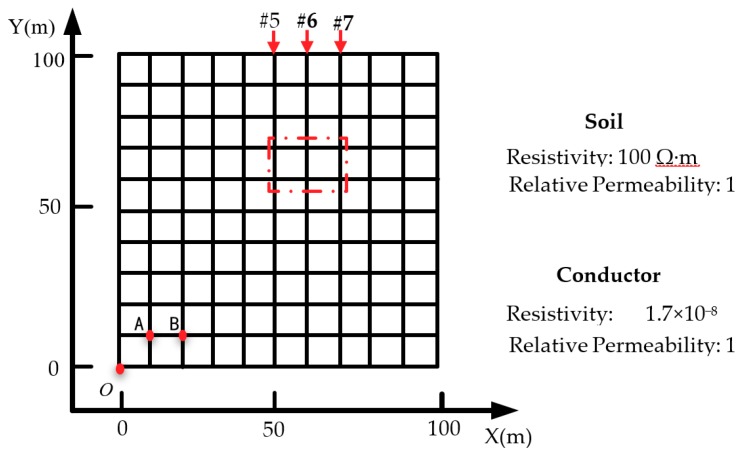
Grounding grid and survey area.

Figure 5 shows a square grounding grid model, which was based on the common grounding nets found in 220 kV substations. The arrangement of the conductors was equally spaced, and the length of each side was 100 m, such that the total area was 100 m × 100 m. Use the point of *o* as the coordinate origin to establish a Cartesian coordinate system. In Figure 5, the red square is the measurement area, #5, #6, #7 is the number of the conductor of the measurement area, and the points A and B are for comparison with the existing injection method, and the current injection points are selected, which will be 3.2, 3.3, respectively, used in the section. 

Copper was selected as the material of the grounding grid conductor and its resistivity is 1.7 × 10^−8^ Ω·m. The diameter of the conductors was 0.0216 m. The grid was buried at 0.6 m below the Earth’s surface. We assumed that the soil was uniform and its resistivity was 100 Ω·m. In addition, the relative permeability of the soil and copper were assumed to be 1, because soil and copper are nonmagnetic materials. 

### 3.2. Simulation Verification of the Four Coil Structure Detection Method

**Figure 6:**
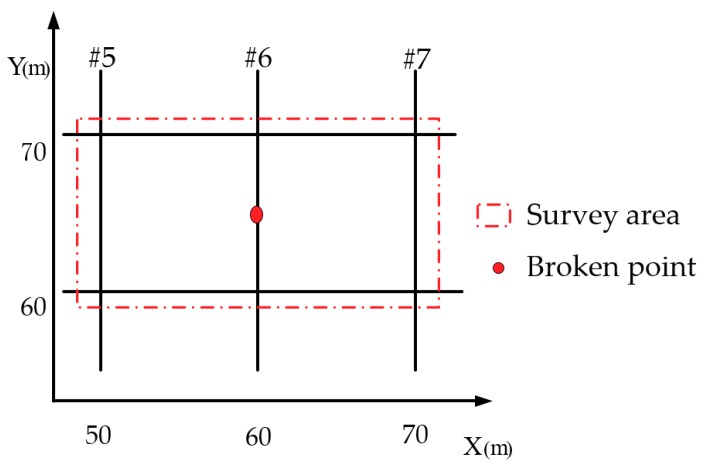
The survey area.

Suppose that the survey area is defined by the red dotted line box in Figure 5. A zoomed-in illustration of this area is depicted in Figure 6. This area consists of three grounding conductors oriented in the *Y* direction and two in the *X-*direction. Twenty-three measuring lines were set from *X* = 49 m to *X* = 71 m. Each measuring line was parallel to the *Y*-axis between *Y* = 59 m and *Y* = 71 m. The interval between the lines is 1 m. Thirteen equally spaced points were placed on each line. The measurement lines at *X* = 50 m, 60 m, and 70 m were oriented along and directly above the grounding grid conductors #5, #6, and #7, respectively. During the measurement, the line between T_x1_ and T_x2_ was always perpendicular to the measuring line, and the midpoint of the line was directly above the measuring point.

**Figure 7:**
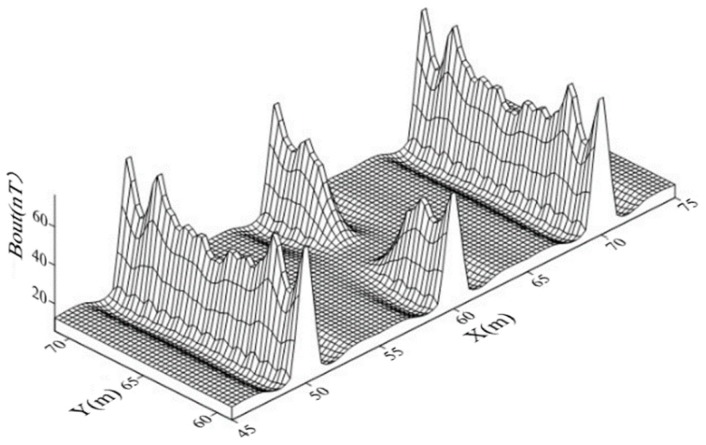
The intensity of the simulated secondary magnetic field $\left(B_{out}\right)$.

Suppose that a breakpoint with a 10 cm gap occurred at the #6 conductor. The distribution of the secondary field magnetic intensity around the measured area is shown in Figure 7. It can be seen from Figure 7 that the intensities of the secondary field, due to magnetic induction, at grounding conductors #5, #6, and #7 were significantly higher than those around the locations where there were no grounding conductors. However, $B_{out}$ at the breakpoint (*X*, *Y*) = (60 m, 65 m) was lower than where the surrounding conductor existed. This phenomenon can be used to determine the locations of the grounding grid conductor and any breakpoints.

### 3.3. Performance Comparison 

A performance evaluation was conducted for the proposed method relative to existing methods in order to demonstrate the advantages of the proposed method. The existing ground fault diagnosis methods for grounding grids based on electromagnetic induction theory were introduced through the grounding grid by injecting and extracting alternating currents [24]. The measurement results were limited by the number and the locations of injection points of the excitation source [3]. The proposed method is a noncontact measurement. The measurement method is not limited by the number and the location of the grounding leads. 

This study used the grounding grid shown in Figure 5 as the simulation object. Two cases of one breakpoint and two breakpoints on the same conductor were discussed. The #6 conductor was selected as the conductor with breakpoints. For the case of a single breakpoint, we set the breakpoint position at *Y* = 65 m, and for the case of two breakpoints, we set the breakpoint positions at *Y* = 65 m and *Y* = 75 m.

#### 3.3.1. Current Injection Measurement Method

**Figure 8:**
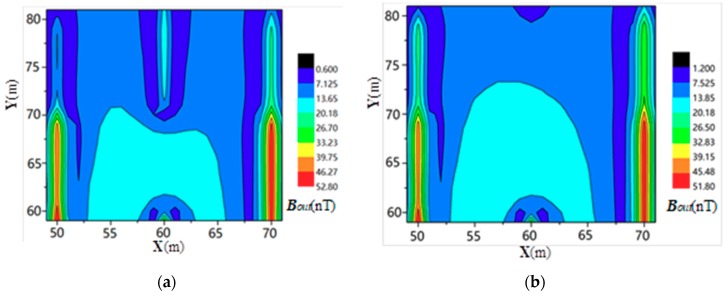
Measurement results of the current injection method [24] for (**a**) one breakpoint and (**b**) two breakpoints.

As shown in Figure 5, Point A (10,10) was selected as the current injection point and point B (20,10) was used as the current extraction point. The distribution of the electromagnetic induction intensity for the current flowing through the ground surface was simulated and calculated. The results are shown in Figure 8. 

#### 3.3.2. Four-Coil Structure Measurement Method

**Figure 9:**
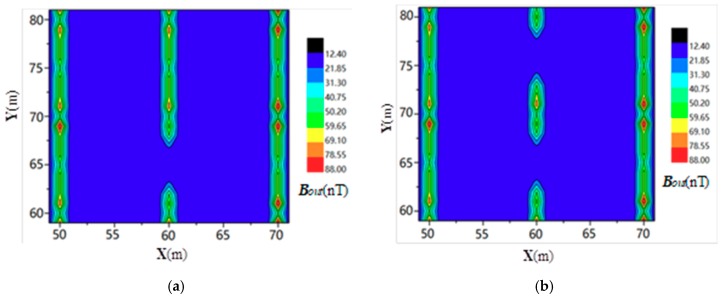
Measurement results from the four-coil structure method for (**a**) one breakpoint and (**b**) two breakpoints.

According to the proposed corrosion breakpoint diagnosis method, the grounding network was simulated and the magnetic field intensity of the secondary field was analyzed. In the test area shown in Figure 6, the simulation was performed based on the above described measurement circuit layout, and the distribution of the secondary field magnetic induction is shown in Figure 9.

Comparing Figure 8 and Figure 9, it is found that when the existing method is used to diagnose the breakage of the grounding grid, the positioning accuracy of the breakpoint(s) is low, since the injection and extraction points of the alternating current are far from the position of the breakpoint(s). It is difficult to distinguish the boundary of the magnetic induction intensity distribution in the area where the point is located. However, the method proposed in this paper is not affected by the number and distribution of grounding wire leads, and thus it can achieve good diagnostic results for any position of the grounding grid. Additionally, the accuracy of the breakpoint positioning is significantly improved. 

Figure 9 shows that the peak of Bout are directly above the conductors and the wave width is about 2 m. The value of Bout in the range of 0 < d < 1 m is significantly higher than the position of *d* > 1 m. At the position of *d* > 1 m, the value of Bout shows no conductor. The main reason for this is because the coupling of the coil to the conductor is reduced during the process of measuring the device away from the conductor to be tested. The transmitting coil spacing set in this paper is 1.4 m, that is, the distance from each coil to the midpoint of the device is 0.7 m. For the measuring point of *d* ≥ 1 m, the two excitation coils move to the same side of the conductor to be tested, and the coupling between the coil and the conductor to be tested is very weak, so *Bout* shows no conductor.

In this section, the geometry of the coil was given, and the distance of the coil is determined by the CDEGS grounding grid simulation software. The simulation of a 100 m × 100 m grounding grid shows that the proposed method can locate the grounding grid conductor and the breakpoint position. In addition, it has also been demonstrated that the method proposed herein overcomes the difficulty of existing methods being limited by ground leads.

## 4. Influence Factor Analysis

The grounding grid is located in a complex environment, and a variety of factors can affect the detection accuracy of the grounding conductors and breakpoints. This paper used the CDEGS software to simulate and discuss the effect of the influence factors on detection accuracy. The influence factors included the number of breakpoints, the excitation frequency, the soil resistivity, and frozen soil caused by seasonal changes.

### 4.1. Effect of the Breakpoint 

Breakpoints may appear at any position of the conductor, such as the midpoint of the conductor branch or the node connecting several conductor branches. To investigate the impact of the position and the number of breakpoints, two cases were considered.

#### 4.1.1. One Breakpoint 

**Figure 10:**
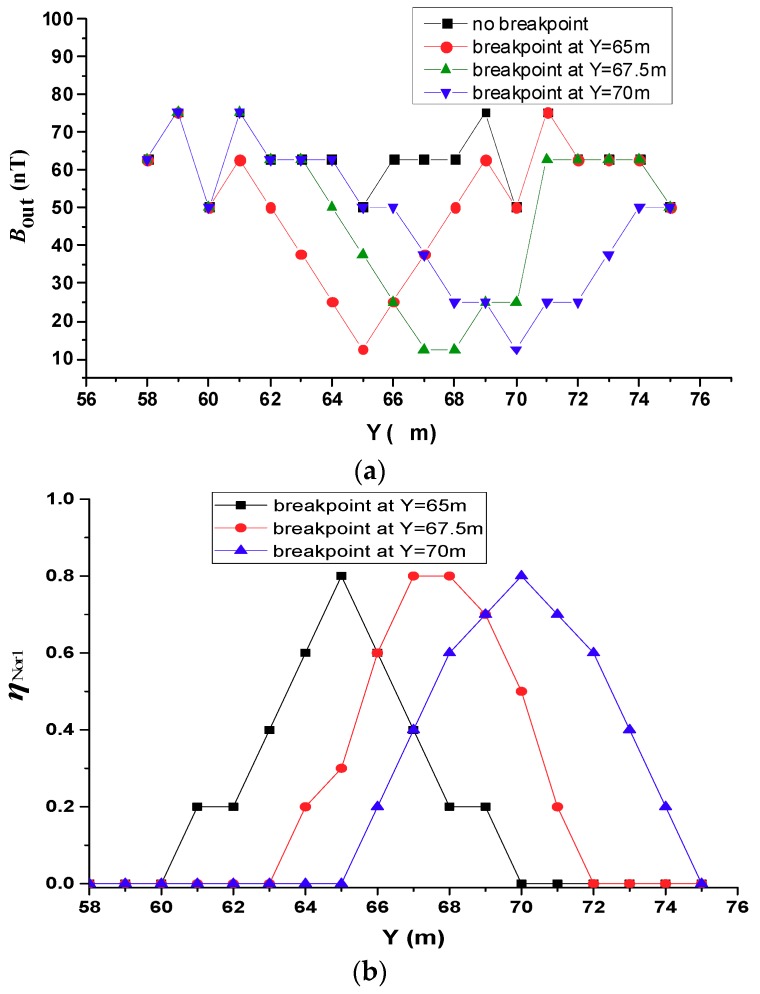
Simulations of one breakpoint occurring at different locations. (**a**) Secondary field along the survey line above the #6 conductor. (**b**) Secondary field changes normalized to the case without one breakpoints.

Consider the setup described in Section 3, we selected the #6 conductor as the object for investigation. Suppose that a single breakpoint with a gap of 10 cm appears at the locations *Y* = 65 m, 67.5 m, and 70 m, as shown in the survey area in Figure 6. Note that these break locations are on the same conductor. The distribution of the magnetic field intensity in the secondary field is along the line directly above the #6 conductor. Figure 10a shows the simulation results of the secondary magnetic field intensity $B_{out}$. Figure 10b shows $\eta_{Nor1}$, the normalized quadratic magnetic field with one breakpoint, shown in Equation (5). This is the simulation results for the secondary magnetic field change as line graph, normalized such that the magnetic field change is measured relative to the case without any breakpoints:

(5) $$\eta_{Nor1}=\left|B_{out\_1}-B_{out\_0}\right|/B_{out\_0}$$

where, $B_{out}$ represents measurement data of the secondary magnetic field. $B_{out\_0}$ means there is no breakpoint, and $B_{out\_1}$ means there is one breakpoint. $\eta_{Nor1}$ represents the normalized quadratic magnetic field with one breakpoint.

It can be observed from Figure 10a that the value of $B_{out}$ at the breakpoint is lower than that at other locations. Figure 10b shows that the relative change of the magnetic field at the location of the breakpoint is significantly higher than the surrounding points. When the measurement point is directly above the breakpoint, the change in the magnetic field at the breakpoint is the largest. When the breakpoint is between two measurement points, the magnetic field strength of the measurement point closest to the breakpoint has the largest change. For points that are far away, the impact of the breakpoint is small. The breakpoint can be clearly located from these profiles regardless of whether the breakpoint was along a conductor branch or at a node connecting grounding conductor branches. The results show that the proposed breakpoint detection method enables accurate localization of a single breakpoint.

#### 4.1.2. Two Breakpoints on the Same Conductor

**Figure 11:**
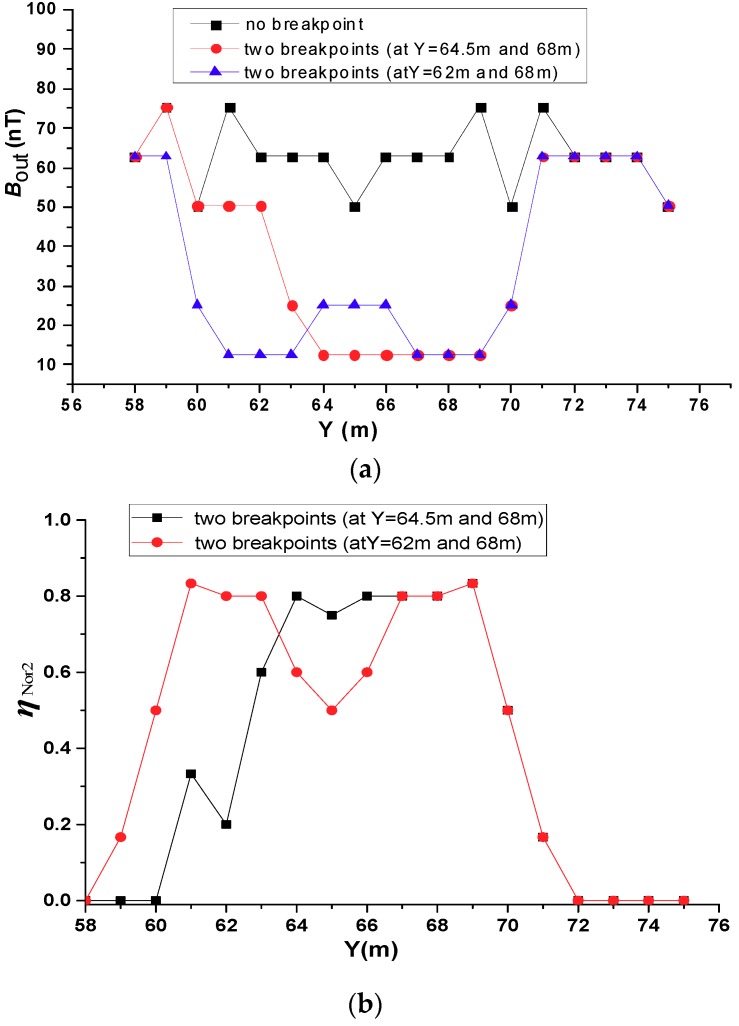
Simulations of two breakpoints at different intervals. (**a**) Secondary field along the survey line above the #6 conductor. (**b**) Secondary field changes normalized to the case without two breakpoints.

In this scenario, we considered two breakpoints on the same conductor. Two situations were considered. In the first case, one breakpoint was set at *Y* = 62 m and another at *Y* = 68 m. These two breakpoints were 6 m away from each other. In the second case, the breakpoint positions were set at *Y* = 64.5 m and *Y* = 68 m. The measurement results are shown in Figure 11a. It can be seen from Figure 11a that the magnetic field intensities directly above the breakpoints are significantly lower than that at other points. When the interval between the two breakpoints is wider, the magnetic field intensities are higher in the middle of the interval. Figure 11b shows the simulation results of the change in the secondary magnetic field changes normalized $\eta_{Nor2}$, it is shown in Equation (6).

(6) $$\eta_{Nor2}=\left|B_{out\_2}-B_{out\_0}\right|/B_{out\_0}$$

where, $B_{out\_2}$ means there are two breakpoints. $\eta_{Nor2}$ represents the normalized quadratic magnetic field with a breakpoint.

When the two breakpoints are far apart, the normalized change in the magnetic field has peaks at the breakpoints. When the two breakpoints are close together, the normalized change in the magnetic field appears as a single broad peak region. Such as when the interval between two breakpoints is 3.5 m, the two breakpoints no longer appear as two independent outliers in the secondary field profile along the survey line. There is still an anomalous region in the field; the magnetic field intensity of the secondary field at the points directly above the two breakpoints and the points between them is the same, and is lower than the secondary field at the other points along the survey line. Although the breakpoints cannot be accurately found when the breakpoint spacing is small, the fact that breakpoints existed could still be determined according to the magnetic field intensity profile, and the number of breakpoints could be roughly determined based on the size of the abnormal region.

Through the above analysis, the following conclusions can be drawn: When a single breakpoint exists, the proposed method can accurately locate the breakpoint, and the positioning accuracy is not limited by the location of the breakpoint. When there are two power failures, the positioning accuracy is affected by the breakpoint spacing. When the spacing is small, the entire area between the two breakpoints exhibits the same low $B_{out}$. When the spacing is large enough, two breakpoints can be accurately located. Furthermore, this method also works for multiple breakpoints.

### 4.2. Effect of the Excitation Frequency

The frequency of the secondary magnetic field induced by the grounding grid conductor is the same as the frequency of the transmitter. The induced current in the conductor is affected by the skin effect. For certain transmission powers, a higher transmission frequency causes stronger impedance in the grounded conductor. This causes more of the induced current to flow along the surface of the conductor where it is easier to diffuse into the soil. As shown in Figure 1, portions of the current flowing into the soil will flow back to the grounding conductor and form a vortex. The higher the frequency, the shorter the vortex path and the higher the resolution. 

**Figure 12:**
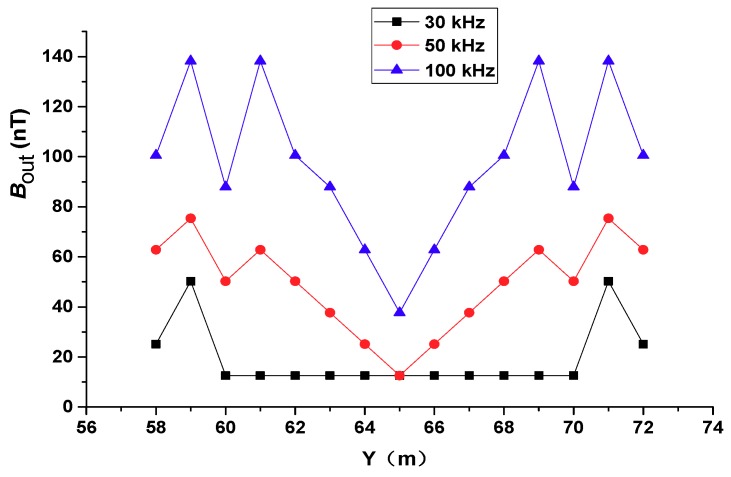
The secondary field intensity along the survey line above the #6 conductor with different emission frequencies.

To analyze the effect of frequency on the detection accuracy of the proposed method, three emission current frequencies were considered: 30 kHz, 50 kHz, and 100 kHz. The breakpoint was set for the #6 conductor at *Y* = 65 m. Figure 12 shows the magnetic induction intensity profile of the secondary field directly above the #6 conductor, and the $B_{out}$ increased with increasing frequency. At 100 kHz and 50 kHz, the location of the breakpoint could be accurately identified. When the frequency was 30 kHz, the $B_{out}$ above the conductor where the breakpoint was located was very low. The table below shows the comparison of the frequency at 50 kHz and 100 kHz. It can be seen from the table that the $B_{out}$ of the measurement point when the excitation frequency was 100 kHz was 1.6–3 times that at 50 kHz. The area adjacent to the breakpoint, *Y* = 65 m, changed significantly.

For low frequencies, the location of the breakpoint on the conductor branch is not clear. Thus, higher frequencies contribute to a more accurate position of the breakpoints. In the above discussion, the amplitude of the transmitting current was constant regardless of the frequency. However, in practice, with an increase of frequency, the impedance of the transmitter coil increases. The maximum voltage and current that the power supply can withstand determines the magnitude of the transmitter current. The magnitude decreases with an increase of the transmitter coil impedance and also influences the intensity of the received signal. In actual operation, the detection accuracy of the breakpoint location and the magnitude of the transmitter current should be considered when determining the frequency.

### 4.3. Effects of Different Soil Resistivities

**Figure 13:**
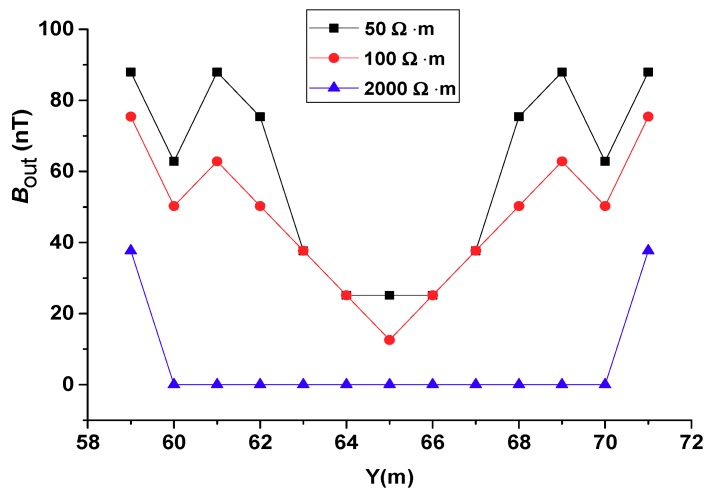
The secondary field intensity along the survey line above the #6 conductor with different soil resistivities.

The soil resistivity range for various soils and rocks varies considerably. In most cases, the soil resistivity where the substation is located varies from tens to thousands of Ω·m. If the soil resistivity is too large, it will affect the dispersion of the soil. Therefore, when the substation is constructed, soils with a small resistivity will be selected. If the soil resistivity is large, the soil will be replaced by a small resistivity soil. Common pastoral soil has a resistivity close to 50 Ω·m, which is an ideal soil that is common in reality. More than 100 Ω·m is basically soil with more sand and stone, and is generally not selected. A range of 50 Ω·m to 100 Ω·m is the soil range that a substation will choose. As 2000 Ω·m is close to the resistivity of the rock, we also considered whether the method proposed in this paper would be effective in this extreme case. We considered three scenarios: Soil with resistivity of 50 Ω·m, 100 Ω·m, and 2000 Ω·m. The breakpoint was set at *Y* = 65 m on the #6 conductor. The length of the gap was 10 cm. The magnetic field intensity of the secondary magnetic field directly above the #6 conductor is shown in Figure 13. It can be seen from Figure 13 that a possible breakpoint is between 64 m and 66 m in the *Y* direction for the 50 Ω·m and 100 Ω·m measurements. For the 2000 Ω·m curve, a breakpoint might exist somewhere within a 10 m range between *Y* = 60 m and *Y* = 70 m. These high resistivity results only show the grounding conductor branch with the breakpoint, but not the exact location of the breakpoint on the branch. In Figure 13, it also shows that $B_{out}$ decreased as soil resistivity increased. The case of 100 Ω·m is about 0.7–0.9 times than that of 50 Ω·m.

The soil resistivity affects the accuracy of the proposed method for the detection of the corrosion breakpoint. For a low resistivity ground, the induced current in the grounding grid conductor can be easily diffused into the ground. Since the flow path of the induced current is short, a high detection accuracy can be achieved. For a high resistivity ground, the dispersion ability of the soil is lessened. The induced current in the primary field mainly flows in the grounding conductor. Thus, the range of the secondary magnetic field is small. This makes it difficult for the receiver coil to detect the secondary field signal at the surface, and thus the detection accuracy is low. 

### 4.4. Effects of the Soil with a High Resistivity Layer 

**Figure 14:**
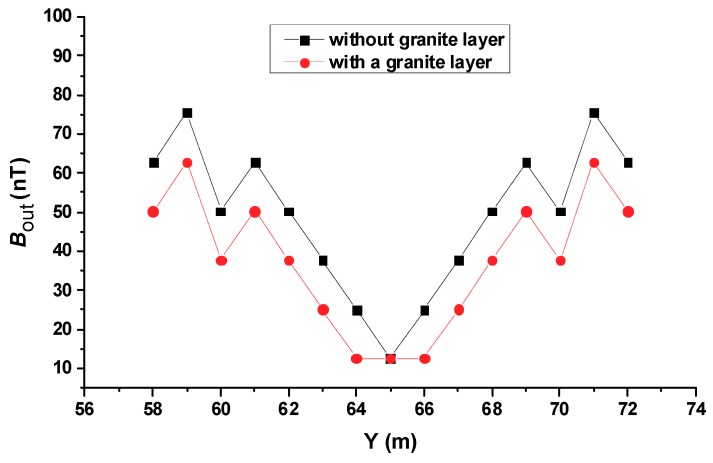
The secondary field intensity along the survey line above the #6 conductor with and without a high resistivity layer present.

As recommended by the IEEE guidelines [1], power facilities such as power plants and substations are required to lay a cover layer of high resistivity material on the surface of the earth to increase the tolerable touch voltage and step voltage. According to the IEEE/PES report [4], the depth of the surfacing layer ranges from 75 to 150 mm and the most minimum wet resistivity specified for the substation surfacing material is 3000 Ω·m in North America. In order to study the influence of a surface-covered high-resistivity layer on the proposed method, two sets of simulations were carried out. In the first group, the soil resistivity was 100 Ω·m and there was no cover on the ground surface. In the second group, a two-layer soil model was designed. The upper layer was granite with a resistivity of 3000 Ω·m. The lower layer contained soil with a resistivity of 100 Ω·m. The thickness of the upper layer was 0.1 m and that of the lower layer was infinite. Table 1 summarizes the two layer model. A current of 10 A and frequency of 50 kHz were the inputs to the transmitter coils. We set a 10 cm breakpoint at *Y* = 65 m on the #6 conductor. The secondary magnetic field profile above the #6 conductor is shown in Figure 14. It can be seen from Figure 14 that the surface layer with a high resistivity only had a slight impact on the detection results. In the case presented in this article, the $B_{out}$ of the cement overlay is about 0.8 times that of the uncovered layer.

### 4.5. Effects of Seasonal Changes

The thickness and the resistivity of a wet or frozen soil layers can vary with seasonal changes [12]. Here, we investigated how the proposed method reacts to such changes. The resistivity of the surface soil layer decreases in the rainy season and increases in the frozen season. The thickness of the surface soil layer affected by the seasonal factor is less than 1.6 m [12]. The soil model established in Reference [12] was adopted in this test. 

A two-layer soil model without a granite cover was considered. The top layer was the soil layer influenced by the seasonal factors, whereas the bottom layer was not affected by the seasonal factors. In general, the resistivity of the seasonal layer varied from 40 to 5000 Ω·m and the thickness was less than 1.6 m. Two cases were considered. Case 1 represents the situation where the thickness of the top frozen layer was 0.4 m. Case 2 represents a 1.6 m thick top layer. The resistivity of the bottom layer in both cases was set as 100 Ω·m. The grounding grid was 0.8 m under the surface. In each case, two situations were considered: (1) No breakpoint in the grounding grid, and (2) one breakpoint on the #6 conductor located at *X* = 60 m and *Y* = 65 m.

The results of the induced magnetic intensity $B_{out}$ of the secondary magnetic field on the surface directly above the breakpoint are shown in Table 2. In Table 2, the top layer of soil is the frozen soil layer and $R_{top}$ is the resistivity. $B_{out}^{0}$ denotes the induced secondary magnetic field intensity when there is no breakpoint, and $B_{out}^{1}$ is the intensity when there is one breakpoint. The difference of these two is $B_{out}^{diff}$, which is used for breakpoint detection. It can be seen from Table 2 that the secondary magnetic field intensity varies significantly when a breakpoint occurs. The difference is greater than 25 nT in all cases. Regardless of the thickness and resistivity of the frozen soil layer, the proposed method can determine if there is a breakpoint in the grounding grid conductor by measuring $B_{out}^{diff}$. Additionally, the results are not affected by seasonal factors.

In this section, various factors that may affect the detection of the grounding grid were considered, such as the number and location of the breakpoints, the frequency of the excitation sources, soil resistivity, the soil with a high resistivity layer, and seasonal changes. The simulation was carried out by CDGES software, which proved that the four-coil grounding grid detection method proposed in this paper can be used in various engineering practical situations.

## 5. Substation Experimental Verification

The four-coil device was designed by the Pro/Engineer operation software, and it is made with polycarbonate plastic. In order to prove the applicability of the device, a substation was selected for field experiment.

### 5.1. Four-Coil Device Design

**Figure 15:**
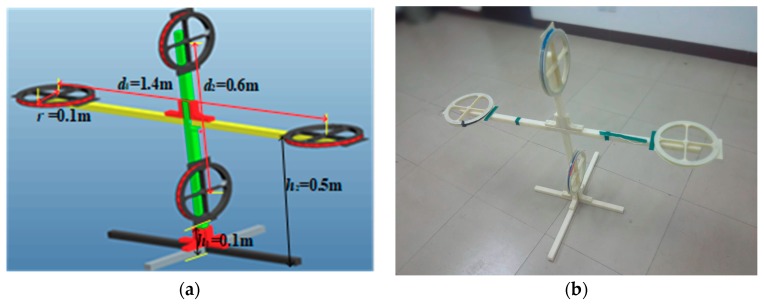
The four-coil device: (**a**) Design diagram and (**b**) finished product.

The four-coil device proposed in this paper was designed by the Pro/Engineer operation software, which is a CAD/CAM/CAE integrated 3D software from the American Parametric Technology Corporation (PTC). The resulting design is shown in Figure 15a. The radius of the four coils was 0.1 m, the transmitting coils were separated by 1.4 m, and the receiving distance were separated by 0.6 m. In order to avoid the influence of ferromagnetic substances on the experiment, the device was constructed using polycarbonate plastic. The finished product is shown in Figure 15b.

### 5.2. Measurement System

**Figure 16:**
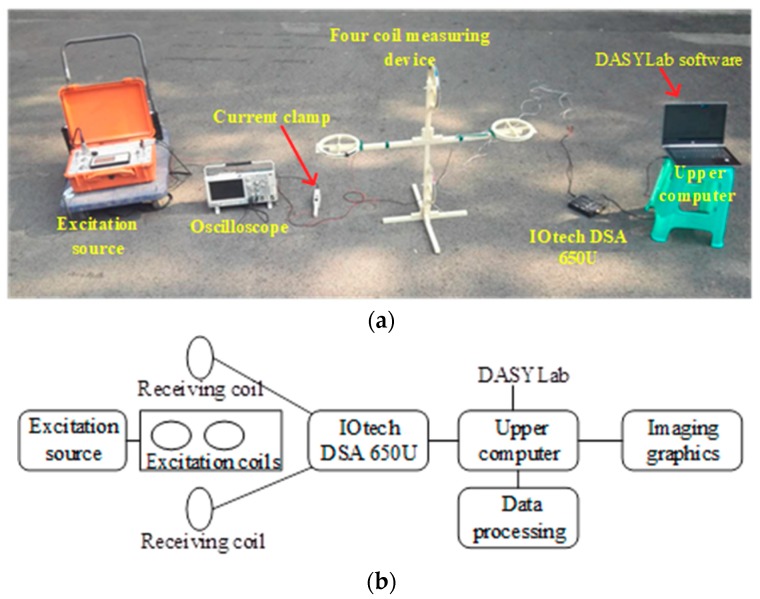
The measurement system: (**a**) Photograph of the equipment wiring for the experimental system; and (**b**) schematic diagram of the experimental process.

The device connection of the measurement system is shown in Figure 16a. The transmitter provides energy for the excitation coil. The transmitter in this experiment was a frequency domain transmitter jointly developed by the authors’ team and Chongqing Triloop Prospecting Technology Co. Ltd. It has not been put into production yet. The research is expected to yield high frequency emission currents in the near future. The four-coil measurement device was proposed and designed by the authors of this paper. The layout and dimensions of the coils were fixed, and the number of turns of the coils could be adjusted as required in engineering practice. In this experiment, each transmitting coil had 15 turns and was made of an enameled copper wire with a radius of 0.25 m, and each receiving coil had 300 turns and was made of an enameled silver wire with a radius of 0.19 mm. The IOtech 650U dynamic signal analyzer transmitted the measured signal from the receiving coil to the host computer. The host computer completed the collection of IOtech upload data through the installed DASYLab data acquisition software. A test was performed to acquire information from the two coils at the same time and ultimately to obtain the magnetic induction intensity of the secondary magnetic field. Thus, two channels in IOtech were used to collect the induced voltage across the two receiver coils. The corresponding grounding grid conductor imaging image was obtained by processing the measurement data. The current clamp and oscilloscope in Figure 16a were auxiliary devices for detecting the transmitted signal and the received signal waveform to ensure that the experiment performed normally. Figure 16b shows a flow chart of data acquisition in the measurement system.

### 5.3. Substation Experimental Verification

**Figure 17:**
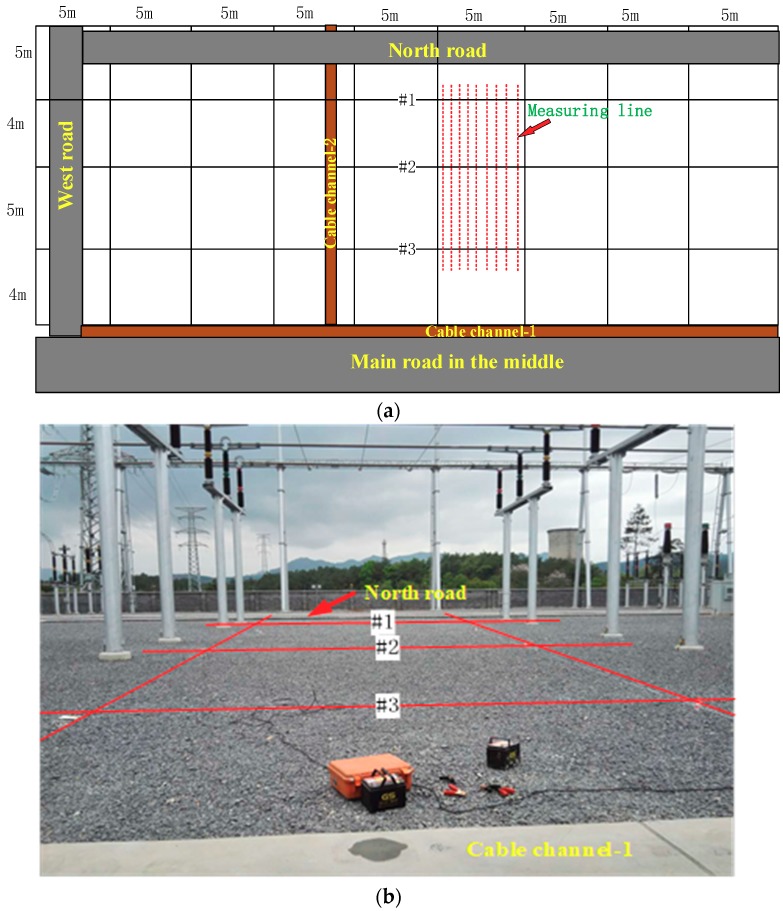
The Chongqing Hongqiang 220 kV substation. (**a**) Topological structure of the grounding grid. (**b**) The experiment site and test field measurement area.

The grounding network of the Chongqing Hongqiang 220 kV substation was selected for the field measurements. The substation has just been built and is not yet in operation, hence it provided a good test environment. Figure 17a shows a schematic diagram of the topology of part of the substation’s grounding grid. The substation longitudinal grounding grid has conductors alternately spaced with separations of 5 m and 4 m. The landscape grounding conductors are evenly distributed with an interval of 5 m. The measurement area is shown in Figure 17a where the red dotted lines indicate the measurement lines. Nine measurement lines were employed with a line spacing of 0.5 m. Each line was 12 m long and 25 measurement points were set, that is, the measuring point spacing was about 0.5 m. The coil was moved to record data at all locations and data at each point were collected for 2 s. The red lines #1, #2, and #3 in Figure 17b correspond to the grounding conductors #1, #2, and #3 in Figure 17a. 

**Figure 18:**
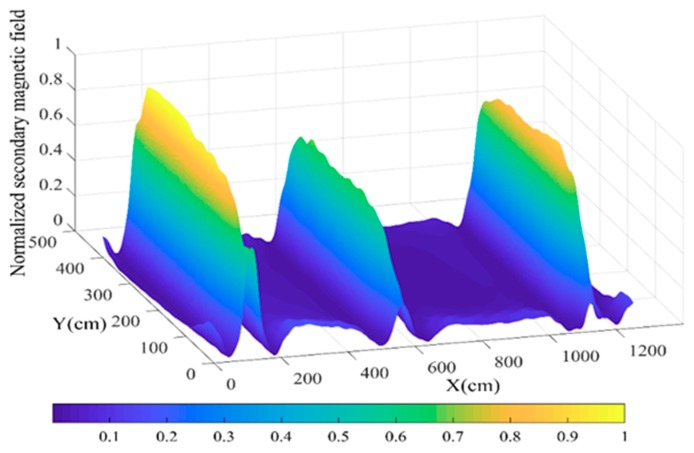
Normalized image of the measurement results for the induced magnetic field.

After processing the measured data, the image of the grounding grid was obtained, as shown in Figure 18. The results show the actual positions of the ground conductors #1, #2, and #3. The amplitude of the secondary magnetic field intensity distributed directly above the conductors of the grounding grid is significantly higher than the position where there is no grounding conductor. 

Figure 18 also shows that the intensities of the three peaks corresponding to the three ground conductors were different. Since the method proposed in this paper is sensitive to metal materials, the metal conductor connected to the grounding grid will affect the measurement structure. There is a cement road (North Road) near the #1 conductor in the north of the measurement area, and a cable channel and a main road near the #3 conductor in the south, as shown in Figure 17. A steel structure was added to the concrete pavement, and the metal casing of the cable trench was connected to the grounding grid, which has an influence on the measurement result. This should be the main reason as to why the peak of the #1 and #3 conductors were greater than that of the #2 conductor.

In this section, the actual grounding grid of the substation was tested experimentally. The measurement results have good agreement with the grounding grid topology design provided by the substation staff. This is shown in Figure 18. The peak was shown directly above the conductor, the width was about 2 m, and the value of *Bout* dropped rapidly within 0–1 m from the conductor. At the position *d* > 1 m from the conductor, the value of *Bout* was equivalent to the position without the conductor. The experimental results were consistent with the simulation results. This is consistent with the simulation results in Section 3, Figure 9. Thus, it is proven that the four-coil grounding grid measurement structure proposed in this paper is feasible and can be applied to grounding network fault state detection.

## 6. Conclusions

This paper presents a method for the fault detection of a substation grounding grid. The proposed scheme involves two transmitter coils and two receiver coils. An induced current flowing in the grounding grid was produced from a primary magnetic field generated by the transmitter loop antennas when the grounding grid conductor segment was in good condition. The secondary magnetic flux density was measured by the receiver loop antennas arranged rationally. The broken section of the grounding grid could be located based on the variation of the secondary magnetic flux density. Compared to other detection methods, the proposed method did not need to inject current into the grounding grid and was not limited by the number and distribution of the grounding leads.

Simulations showed that the four-coil structure detection method proposed in this paper could be applied to the grounding grid state detection under various conditions. The CDEGS grounding network simulation software was used to simulate various factors that may affect the diagnosis of grounding network corrosion breakpoints, and the following conclusions were obtained:

1) The proposed method can locate the conductor breakpoints. When there is only one breakpoint, regardless of where it is, it can be located accurately. When there are two breakpoints, the proposed method can obtain precise positioning of distant breakpoints. However, if the two breakpoints are close to each other, then their area can be located.
2) The signal resolution for the breakpoint detection of the proposed method is affected by the frequency of the emission current. A higher frequency contributes to more accurate detection.
3) The effect of soil resistivities were analyzed. The proposed method works better at lower soil resistivity, but when the resistance rate was as high as 2000 Ω·m, it could still be applied.
4) The simulation results show that a soil layer of large resistivity has little influence on the capabilities of the proposed method.
5) The influence of seasonal changes was studied. Although seasonal variation can affect soil resistivity, it does not affect the detection accuracy of this method.

Experiments were carried out on a substation using the four-coil device studied in this paper. The experimental results show that the location of the grounding conductor can reasonably explain the actual situation of the substation, which effectively proves that the method can be applied to grounding grid detection.

## Figures and Tables

**Table 1:** Soil model with a granite layer.

Layer	Resistivity (Ω·m)	Thickness (m)
Top	3000	0.1
Bottom	100	infinite

**Table 2:** The induced secondary magnetic field intensity $B_{out}\left(\mathrm{nT}\right)$ measured on the surface at point (60 m, 65 m).

	Cases 1 (0.4 m)			Cases 2 (1.6 m)		
$R_{top}$ (Ω⋅m)	$B_{out}^{0}\;(\mathrm{nT})$	$B_{out}^{1}\;(\mathrm{nT})$	$B_{out}^{diff}\;(\mathrm{nT})$	$B_{out}^{0}\;(\mathrm{nT})$	$B_{out}^{1}\;\left(\mathrm{nT}\right)$	$B_{out}^{diff}\;(\mathrm{nT})$
40	50.26	12.57	37.69	62.83	25.13	37.70
100	37.70	12.57	25.13	37.70	12.57	25.13
200	37.70	12.57	25.13	37.70	0.00	37.70
500	37.70	12.57	25.13	25.13	0.00	25.13
1200	37.70	12.57	25.13	25.13	0.00	25.13
5000	37.70	12.57	25.13	25.13	0.00	25.13
